# Feedback on Escape Behavior of Birds Under Different Hunger Pressure

**DOI:** 10.1002/ece3.70866

**Published:** 2025-01-21

**Authors:** Xiao‐Yang Bao, Jia‐Jia Xin, Yuan‐Xing Ye, Can‐Shi Hu

**Affiliations:** ^1^ College of Life Sciences, Guizhou University Guiyang Guizhou China; ^2^ School of Ecology and Nature Conservation, Beijing Forestry University Beijing China; ^3^ Research Center for Biodiversity and Nature Conservation Guizhou University Guiyang Guizhou China

**Keywords:** behavior strategy, energy trade‐off, flight initiation distance, hunger pressure, vigilance behavior

## Abstract

The risk of predation has always been a significant impact on wild birds. Birds, facing with limited energy, must balance their investment between foraging and vigilance. There were currently limited understandings of the vigilant behavior feedback of birds under different hunger pressure. Therefore, we employed the White‐browed laughingthrush (*Pterorhinus sannio*) and White wagtail ( *Motacilla alba* ) as research subjects to carry out experiments in winter, in exploring the tolerance of birds to external stress under different hunger pressure. After a night of energy expenditure, individuals of both species faced greater hunger pressure in the morning. The results of general linear models showed that the flight initiation distance (FID) of both species in the morning (7:00–9:00) was significantly shorter than that in the evening (16:00–18:00). Additionally, when the weather was cold (daily minimum temperature ≤ 5°C), the FID of the White‐browed laughingthrush and White wagtail was significantly shorter in the morning, as same as the results of general linear models. However, when the weather was warm (daily minimum temperature ≥ 10°C), there was no significant difference even though the FID average of both species was shorter in the morning than in the evening. These suggested that the consumption and supplementation of energy are very important for birds, as the higher their hunger pressure, the greater their willingness to forage and take on risk, especially in cold winter.

## Graphical abstract

The balance between risk and benefit for birds in the face of external stress is highly significant. Our research has revealed that birds with heightened hunger pressure were more inclined to undertake greater risks when confronted with external stress, consequently increasing their foraging opportunities.

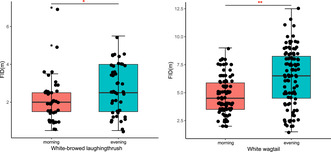

## 1 Introduction

Predation risk has always presented significant challenges to animal survival (Anholt and Werner 1998; Cukor et al. 2024). It can temporarily disrupt animal activities, and in severe cases, result in injury or death (Janson 1998). Even animals that are rarely preyed upon can be significantly affected by the risk of predation, influencing their behavior (Lima and Dill 1990). Failing to evade predators during a specific excursion can have catastrophic consequences for individual animal survival. Predation risk can induce antipredation behaviors, including animal vigilance (Bouskila and Blumstein 1992). Consequently, vigilant behavior in animals is crucial for species survival (Sansom, Lind, and Cresswell 2009). For birds, the purpose of vigilance is to prevent the interests of individuals from being harmed. When birds encounter external threats like predators, their stress response typically involves escape and adaptation (Sansom, Lind, and Cresswell 2009). Escape is an important strategy for birds to avoid predation. However, this behavior will cause additional energy consumption, including energy expenditure during flight and insufficient energy acquisition due to reduced foraging opportunities (Nudds and Bryant 2000). Flight initiation distance (FID) is frequently used to measure escape behavior in birds (Cooper and Blumstein 2015). The straight‐line distance between the predator and the prey when the prey initiates escape is called FID.

Vigilance level represents a trade‐off between acquiring enough food and evading predators (Kaitala, Lindström, and Ranta 1989). Enhancing bird foraging success can increase individual survival rates (Cresswell et al. 2003; Sansom, Lind, and Cresswell 2009). Previous studies have demonstrated the reciprocal relationship between bird alertness and foraging (Lendrem 1983; Popp 1988). Generally, when a predator approaches from a distance, the foraging bird will not immediately flee (Clark 1994). Instead, it will choose to continue foraging until the predator is very close to it before escaping. Through the optimal escape theory and the economic theory, individuals will choose to flight when the risk of survival is greater than the cost of escape (Cooper Jr and Frederick 2007; Ydenberg and Dill 1986). However, the theories does not explore escape behavior from the perspective of hunger pressure. As we all know, small birds have the characteristics of large flight energy consumption and high basic metabolism. Especially on cold winter nights, birds will consume a lot of energy in order to maintain body temperature (Blem 1976), so it can be predicted that the hunger pressure is greater in the morning. In theory, the birds with high hunger pressure would intensify their food motivation (Houston, McNamara, and Hutchinson 1993; McNamara and Houston 1992). Hence, hunger pressure may influences the feedback of birds escape behavior, that is, the more hunger pressure the birds is, the later it will escape.

For small birds, they will experience heightened hunger after a night of fasting and energy expenditure (Reinertsen and Haftorn 1986). Especially in the cold winter, birds need to consume more energy at night to maintain a constant body temperature (Blem 1976). Subsequently, as hunger intensifies the next morning, birds require more energy and engage in increased foraging (Sih 1987), leading to a greater willingness to assume risks. Simultaneously, the scarcity of food resources in winter diminishes bird foraging opportunities, causing birds to tolerate higher risks when confronted with external threats like predators while foraging. Post‐morning feeding, the hunger pressure on birds diminishes in the afternoon, consequently reducing their willingness to take risks. To explore if hunger primarily contributes to increased tolerance in birds, we further speculate that birds require additional energy expenditure to regulate their body temperature in extremely low nighttime temperatures. Therefore, in the morning of the next day, they will have a stronger sense of hunger and are willing to bear greater risks. Consequently, on cold days, the disparity in FID between morning and evening becomes more pronounced, indicating that birds exhibit greater tolerance when experiencing heightened hunger pressure. As we utilized FID as a quantitative measure of bird escape behavior, we made the following two sets of predictions about how hunger pressure affects the FID values of birds: (1) After a night of fasting, the energy consumption is greater, and birds should have greater hunger pressure in the next morning. Compared to the evening after foraging, birds should have lower FID values in the morning. In other words, when the hunger pressure is high, birds will take a greater risk of predation in order to obtain more foraging opportunities. (2) Birds expend more energy to maintain body temperature at night when temperatures are colder, which in turn leads to higher hunger pressure in the next morning. Therefore, the difference in the FID values of birds between morning and evening is greater.

## 2 Methods

### 2.1 Study Site

Our experiment was carried out in the campus of Guizhou University (26°27′ N, 106°39′ E) that is situated in Huaxi District, Guiyang City, Guizhou Province, China. Guizhou University belongs to the subtropical plateau monsoon humid climate area, which has the characteristics of pleasant climate, adequate rainfall and abundant biological resources. The region experiences an average winter temperature of 6.6°C, with the lowest temperature recorded at −4.2°C. The habitat of the campus is dominated by open areas such as lawns, hardened roads and squares. There are many kinds of birds on campus, with the White‐browed laughingthrush (*Pterorhinus sannio*), and White wagtail ( *Motacilla alba* ) being the dominant species.

### 2.2 FID Measurement

The experiment was conducted throughout the winter period from November 2022 to January 2023. The research focused on the White‐browed laughingthrush and White wagtail as the subjects, and their escape behavior was quantified using the FID. The White‐browed laughingthrush and White wagtail are widespread species with large populations, which were easier to observe during the experiment. The White‐browed laughingthrush usually forages on the ground around shrubs, and when in danger, it will fly or run to the base of the shrubs, shuttle or hide among low branches (Zhao 2001). The White wagtail prefers to foraging on the ground, and will run away or fly when facing with danger (Zhao 2001).

The study implemented a natural fasting model (Castellini and Rea 1992). Since the White‐browed laughingthrush and White wagtail fasted during the night, it was considered a natural form of hunger treatment for them. After a night of fasting and energy expenditure, both species would have greater hunger pressure in next morning. In the evening, after a whole day of feeding, the hunger pressure of birds was lighter than in the morning (Bednekoff and Houston 1994; Lendvai et al. 2004). It is important to note that the experiment was conducted during the colder winter months. So, compared to other seasons, birds needed to spend more energy to maintain a constant body temperature at night and was more likely to experience greater hunger on winter mornings.

Prior to commencing the experiment, researchers needed to practice walking at a average speed since the approaching speed affects the FID of birds (Bateman and Fleming 2011; Hammer et al. 2022). Only individuals foraging on open ground were selected as research subjects to mitigate the impact of microenvironmental variation (Hall et al. 2020). Birds active in areas such as shrubs or branch were not chosen as research subjects to minimize the impact of vertical height (Blumstein et al. 2004). During the experiment, researchers wore black or gray clothing to minimize the influence of brightly colored attire (Jiang, Liang, and Zhang 2023), and chose good weather conditions with sunrise, no rain, and few clouds for the survey (Hammer et al. 2022), with two time periods: morning (07:00–09:00, according to the Beijing Time, hereafter) and evening (16:00–18:00). After observing the research subjects during foraging, we approached them at a consistent speed of ca. 0.4 m/s until they left the foraging area and stop moving. We then used a laser rangefinder (Onick 1200 L) to measure the straight‐line distance between the researcher and the flight location, which was recorded as the flight initiation distance (FID).

The FID of birds could be also influenced by other factors. In this study, the group size and number of humans were identified as the main factors influencing FID (Beauchamp 2008). A cluster was defined as a group of two or more individuals, and one was randomly selected for observation. Number of humans referred to the number of people present within a 20 m radius of the observed subject during the measurement process. In field experiments, it is possible to observe the same individual repeatedly, so the White‐browed laughingthrush and White wagtail, with large populations, were chosen as research objects. Due to birds don't fly long distances in short periods of time (Odum and Kuenzler 1955), we observe in different areas, with adjacent locations larger 500 m, at different times of the day. Moreover, we also extended the interval of the observations, and made another observation every 3 days.

We recorded the daily minimum temperature that represents the lowest temperature at any time during the 24 h of the day (0:00–23:59), and studies have shown that the daily minimum temperature of the day is usually in the morning before sunrise (Reicosky et al. 1989). The FID values of the White‐browed laughingthrush and White wagtail were categorized into two groups based on the daily minimum temperature: high hunger pressure group (daily minimum temperature ≤ 5°C) and low hunger pressure group (daily minimum temperature ≥ 10°C). When night temperatures were colder, birds used up more energy reserves to maintain body temperature (Blem 1976), and they faced greater hunger pressure in the next morning. Therefore, the daily minimum temperature was selected to reflect the cold level at night. During our investigation, the daily minimum temperature was mainly distributed in the two ranges of −2°C–5°C and 10°C–17°C, and the two ranges represent colder nights and warmer nights respectively. When the night temperature was low, the hunger pressure of birds in the morning was higher, and the opposite was true when the night temperature was high.

### 2.3 Statistical Analysis

In order to investigate the effects of all variables (time period, group size and number of humans) on FID, we put them into a General Linear Model (GLM) for analysis, and the two species were modeled separately. We use variance inflation factor (VIF) to evaluate the multicollinearity of the model (Akinwande, Dikko, and Samson 2015). Studies have shown that when VIF > 5, the multicollinearity of the model has a high correlation (Daoud 2017). We used a full subset model with the Akaike information criterion (AIC) to rank the optimal model (Hu 2007). Due to the existence of multiple approximate fitting models (△AIC ≤ 2), the “average model” of the R package “*MuMIn*” calculation was used for summarization (Barton and Barton 2015). Meanwhile, in order to demonstrate the importance of each variable to FID, we used the “importance” function in the R package “*MuMIn*” to calculate their weights, which were considered “important” when greater than 0.7 (Nacif, Quintero, and Garibaldi 2021).

Based on the model results, we used the R package “ggplot2” to draw box plots of the impact of two species on FID at different time periods. Considering that the FID of White wagtail was influenced by two variables, period and number of humans (see “Results”), we used the R package “visreg” to draw a partial correlation graph of the impact of number of humans on its FID (Breheny and Burchett 2017). The data was presented as “Mean ± SD”, and statistical significance was determined when *p* < 0.05. All the aforementioned analyses were performed using R4.2.3 statistical software.

## 3 Results

**FIGURE 1:**
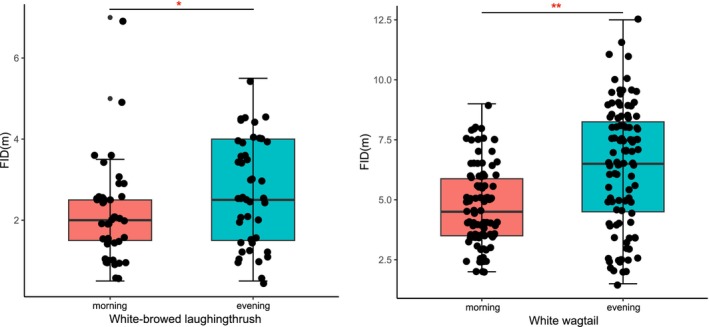
The difference of FID of White‐browed laughingthrush (*Pterorhinus sannio*) and White wagtail ( *Motacilla alba* ) in the morning and evening respectively. Significant differences were marked with either *p < 0.05 or **p < 0.01. The upper and lower whisker respectively denote the maximum and minimum thresholds of the FID, with data points falling outside these limits typically regarded as outliers.

**FIGURE 2:**
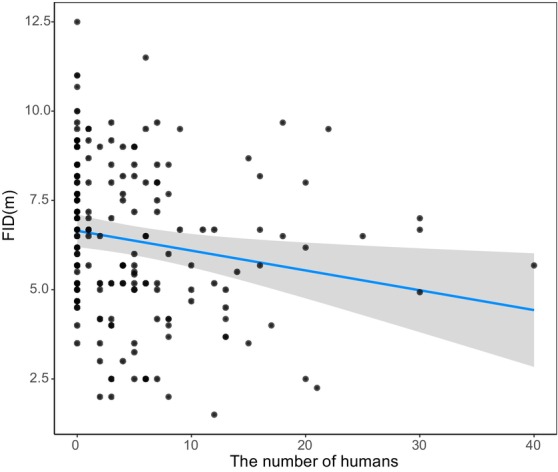
The relationship between number of humans and FID of White wagtail ( *Motacilla alba* ). The “blue line” represents the fitting line, and the “gray area” represents the 95% confidence interval.

In this study, the White‐browed laughingthrush was collected 42 and 45 observations in the morning and evening, and the White wagtail was collected 102 and 103 observations in the morning and evening, respectively. The VIF indicates that there was no multicollinearity between the different variables (Range: 1.000–1.027). The statistical results of the general linear model showed that the FID of White‐browed laughingthrush (*z* = 2.052, *p* = 0.040, Table 1) and White wagtail (*z* = 5.706, *p* < 0.001, Table 1) had significant differences in different time periods. In terms of significance, the relative importance values of the various factors showed similar results (Table 2). The FID in both species was shown to be shorter in the morning (Figure 1). Number of humans had a negative significant correlation on the FID of White wagtail (*z* = 2.501, *p* = 0.012, Table 1). With the increase of number of humans, FID decreases gradually (Figure 2).

**TABLE 1:** Results of the average model explaining flight initiation distance (FID) throughout the morning and evening in White‐browed laughingthrush (*Pterorhinus sannio*) (*n* = 87) and White wagtail ( *Motacilla alba* ) (*n* = 205).

Dependent variable	Species	Independent variable	Coefficient	SE	*z*	*p*
FID	White‐browed laughingthrush	Intercept	2.113	0.225	9.256	﹤ 0.001
		Period: evening	0.560	0.269	2.052	0.040
		Group size	−0.001	0.018	0.047	0.962
		Number of humans	0.008	0.023	0.339	0.735
	White wagtail	Intercept	4.864	0.322	15.048	﹤ 0.001
		Period: evening	1.680	0.293	5.706	﹤ 0.001
		Group size	0.068	0.145	0.469	0.639
		Number of humans	−0.056	0.022	2.501	0.012

**TABLE 2:** The relative importance values of the various factors affecting the FID of the White‐browed laughingthrush (*Pterorhinus sannio*) and White wagtail ( *Motacilla alba* ) in the average model.

	Period	Group size	Number of humans
White‐browed laughingthrush	**1.00**	0.20	0.27
White wagtail	**1.00**	0.37	**1.00**

*Note:* Factors with relative importance values greater than 0.70 (marked in bold) are considered important to the model set.

**FIGURE 3:**
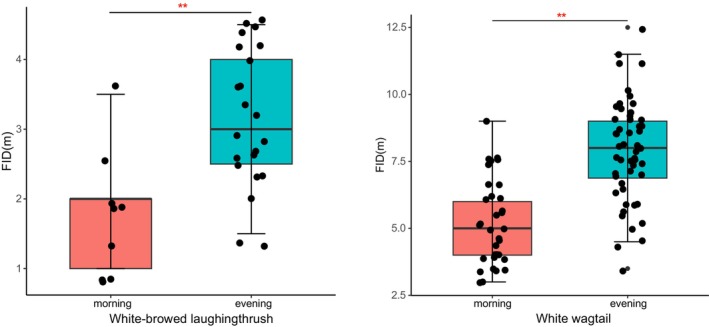
The difference of FID of White‐browed laughingthrush (*Pterorhinus sannio*) and White wagtail ( *Motacilla alba* ), in the morning and evening when the minimum daily temperature was less than or equal to 5°C, respectively. Significant differences were marked with **p < 0.01. The upper and lower whisker respectively denote the maximum and minimum thresholds of the FID, with data points falling outside these limits typically regarded as outliers.

In the low hunger pressure group, the White‐browed laughingthrush was collected 33 and 23 observations in the morning and evening, and the White wagtail was collected 69 and 51 observations in the morning and evening, respectively. There was no significant difference between the morning and the evening for White‐browed laughingthrush (*p* = 0.150) or White wagtail (*p* = 0.299), although it's shorter in the morning than that in the evening for FID of White‐browed laughingthrush and White wagtail. In the high hunger pressure group, the White‐browed laughingthrush was collected 9 and 22 observations in the morning and evening, and the White wagtail was collected 33 and 52 observations in the morning and evening, respectively. There was a significant difference between the morning (1.83 ± 0.83 m) and the evening (3.16 ± 0.96 m) of White‐browed laughingthrush (*p* < 0.001, Figure 3), and there was a significant difference between the morning (5.20 ± 1.56 m) and the evening (7.88 ± 1.86 m) of White wagtail too (*p* < 0.001, Figure 3).

## 4 Discussion

Our study investigated the changes in the FID of the White‐browed laughingthrush and White wagtail under different hunger pressures. Due to the fact that the research subjects will not eat at night, the next morning is the time when the individual hunger pressure is the highest, and the FID of the two species is significantly shorter than that of the low hunger pressure. The shorter FID of birds means that they can get more food, so they can get more energy intake, which may be the best escape strategy after energy trade‐off. The colder nights in winter will increase the energy consumption of birds, which will expand the difference of hunger pressure between in the morning and evening. Our results showed that the FID of high hunger pressure group (Daily minimum temperature ≤ 5°C) and low hunger pressure group (Daily minimum temperature ≥ 10°C) were shorter in the morning, but only the FID of high hunger pressure group was significantly different in the morning and evening.

As we predicted, the FID of the White‐browed laughingthrush and White wagtail was shorter in the morning. This may be the result of birds weighing energy and safety requirements, consistent with the optimal escape theory (Cooper Jr and Frederick 2007). In the morning, under the highest hunger pressure, the birds may have considered it necessary to reduce unnecessary energy expenditure. Flight will cause a lot of energy consumption (Nudds and Bryant 2000), so birds under high hunger pressure will not choose to fly away from predators at the first time. In contrast, instead of flying away immediately, the bird will choose a strategy that is weigh flying or not flying, and it will be forced to fly away only when it believes the threat is greatest. In nature, the appearance of some predators does not mean that they must hunt prey, and this strategy may make birds facing high hunger pressure reduce unnecessary flight, so as to save energy. Research on nectarivorous birds with high metabolism and low energy storage has also found that the New Holland honeyeaters ( *Phylidonyris novaehollandiae* ) chose a lower energy consumption escape strategy that compared with escape strategy (flying), escape strategy (running) saved more energy and could quickly return back (Ferguson, Gilson, and Bateman 2019). This strategy of increasing the risk of predation to obtain more energy is not unique to birds, and mammals will also adopt similar strategies. A study on ground squirrels ( *Spermophilus dauricus* ) showed that, when under higher hunger pressure, they opted for more energy‐efficient vigilance behaviors in order to reduce energy expenditure, even though this strategy greatly increases their probability of being preyed upon. (Shuai et al. 2022).

Foraging opportunities may be very important for birds that are under great hunger pressure. After discovering predators, birds will balance their foraging and escaping based on their own conditions. If birds escape too early, they will lose foraging opportunities; while if they escape too late, they will greatly increase the risk of predation (Møller, Vágási, and Pap 2013; Tatner and Bryant 1986). When not affected by hunger pressure, birds will immediately escape after observing their natural enemies, and are willing to give up more foraging opportunities in the face of external threats to ensure their own safety. The economic theory suggests that birds typically weigh the costs of continuing to forage and escape, in order to maximize their own interests (Ydenberg and Dill 1986). Our conclusion is consistent with the economic theory, that is, when facing with enormous hunger pressure, the White‐browed laughingthrush and White wagtail did not immediately stop their foraging activities in the face of external stress, but instead delayed their escape to obtain more foraging opportunities. This will minimize the relative cost of birds staying and escaping. Research on Hooded crows ( *Corvus cornix* ) has also found that when it is foraging for profitable food, it will delay its escape time in the face of external pressure and expand its food gain (Novčić 2023).

Previous studies have shown that the FID was influenced by human interference, and our research has also confirmed this. Our results indicated that with increasing number of humans, the FID of the White wagtail became shorter. This may be related to our experiment conducted in urban ecosystems, where birds have a certain tolerance. In the process of rapid urbanization, the birds that stay in cities can quickly adapt to urban environments and settle down. Comparing with the same species that inhabit rural areas, the bird population living in cities has higher tolerance (Kathryn and Sarah 2016; Yin et al. 2023). Human intruders, the number of people appearing within a certain range of bird activity, is an important indicator affecting birds' tolerance (Burger and Gochfeld 1991; Erwin 1989). Research on the Great crested grebe( *Podiceps cristatus* )showed that as the intensity of human intruders increases, its flight initiation distance became shorter and its tolerance to external stress increased (Keller 1989). The tolerance of the White wagtail to human intruders also showed an increasing trend.

Our study also confirmed some previous studies that found differences in escape behavior among different bird species (Burger 1981; Blumstein et al. 2003). Our results showed that White‐browed laughingthrush with larger body size will choose to fly away later when faced with external threats (Figure 1). This may be related to the number of food requirements. Some studies have found that larger birds tended to require more food supplementation and generally did not choose to leave their foraging grounds immediately unless they must (Glover et al. 2011). In addition, we found an interesting phenomenon that hunger pressure may have different effects on FID in birds of different body sizes. The FID variability of White wagtail was higher under different hunger pressures (Figure 3). It may be that White wagtail has a smaller body size, relatively less energy storage, and is more likely to be hungry or full. Therefore, compared with normal conditions, when the hunger pressure is greater, the FID of birds with small body size may have greater elasticity.

There are some limitations in this study. In field experiments, we are unable to mark and identify the individuals, so the probability of repeated observations can be reduced by increasing the sample size, expanding the research scope and interval. Therefore, the conclusion of our study still has a high credibility. It is not only hunger pressure that affects escape behavior, but also predation pressures in different regions may affect it (Shuai et al. 2022). Studies have shown that prey living under high predation pressure will flee earlier when faced with external threats (Diego‐Rasilla 2003). No predators were observed throughout our experiment, so we think that the predation pressure our subjects face on campus is consistent. In addition, there may be differences in food motivation between White‐browed laughingthrush and White wagtail at different periods in the morning and evening (Bonter et al. 2013). Food motivation is one of the indicators to measure the hunger pressure of animals. Individuals who showed higher food motivation, faced higher hunger pressure (Munk 1995). However, our research objects are active during the day and rest at night, most individuals may face greater hunger pressure in the morning than in the evening. So, food motivation didn't have much impact on our results.

## 5 Conclusions

The study found that the birds' decision to escape or willingness to take risks was influenced by the hunger pressure and the number of humans. Through this study, the correlation between hunger pressure and escape behavior of birds was further explored. The results showed that the escape behavior of birds under high hunger pressure takes into account the minimization of energy consumption and the increase of foraging opportunities. Future research could explore how escape behavior changes in birds of different body sizes under different hunger pressures.

## Author Contributions

**Xiao‐Yang Bao:** conceptualization (equal), data curation (lead), formal analysis (equal), investigation (lead), methodology (equal), writing – original draft (lead). **Jia‐Jia Xin:** conceptualization (equal), data curation (supporting), writing – review and editing (supporting). **Yuan‐Xing Ye:** conceptualization (equal), data curation (supporting), formal analysis (equal), methodology (equal), writing – review and editing (supporting). **Can‐Shi Hu:** conceptualization (equal), formal analysis (equal), methodology (equal), project administration (lead), writing – review and editing (lead).

## Conflicts of Interest

The authors declare no conflicts of interest.

## Data Availability

Editors and reviewers have access to all necessary data files.
